# Role of Tocilizumab in the Treatment of COVID-19 Patients with Cytokine Storm: A Case Series

**DOI:** 10.31729/jnma.7364

**Published:** 2022-08-31

**Authors:** Ajaya Basnet, Mahendra Raj Shrestha, Rossu Thapa, Basanta Tamang, Apurba Shrestha, Prabhat Rawal, Sailendra Kumar Duwal Shrestha, Lochan Karki, Shiba Kumar Rai

**Affiliations:** 1 Department of Medical Microbiology, Shi-Gan International College of Science and Technology, Maharajgunj, Kathmandu, Nepal; 2 Department of Clinical Laboratory, Nepal Armed Police Force Hospital, Balambu, Kathmandu, Nepal; 3 Department of Radiology, Nepal Armed Police Force Hospital, Balambu, Kathmandu, Nepal; 4 Department of Internal Medicine, Nepal Armed Police Force Hospital, Balambu, Kathmandu, Nepal; 5 Department of Anaesthesiology and Critical Care, Nepal Armed Police Force Hospital, Balambu, Kathmandu, Nepal; 6 Department of Orthopedic and Trauma Services, Nepal Armed Police Force Hospital, Balambu, Kathmandu, Nepal; 7 Department of Medicine, National Academy of Medical Sciences, Bir Hospital, Mahaboudha, Kathmandu, Nepal; 8 Research Department, Nepal Medical College and Teaching Hospital, Jorpati, Kathmandu, Nepal

**Keywords:** *COVID-19*, *cytokines*, *interleukin-6*

## Abstract

The in-hospital mortality in patients with COVID-19 could be correlated with severe acute respiratory syndrome coronavirus-2 induced hyper-inflammation, which is attributed to an unconstrained inflammatory cytokine storm. The pro-inflammatory cytokine, specifically, interleukin-6 plays a prominent role in the cytokine storm and may result in alveolar-capillary blood-gas exchange dysfunction. Therefore, the method to block the signal transduction pathway of interleukin-6 could be a potential treatment for severe COVID-19 patients. In this case series of three patients with severe COVID-19, we focus on the rationale for utilization of tocilizumab, an anti-interleukin-6 receptor antibody, which could block the signal transduction pathway of interleukin-6. The observations from this study allowed us to hypothesize that the infusions of tocilizumab may not reduce the elevated level of interleukin-6, and hence may not be a significant therapeutic for reducing in-hospital mortality associated with COVID-19. Additionally, it could also be speculated that interleukin-6 may not be a potentially actionable target cytokine to treat COVID-19-associated cytokine storms.

## INTRODUCTION

The binding of proinflammatory interleukin-6 (IL-6) to the IL-6 receptor, which is present on the host's target cells, leads to the cytokine storm in patients with COVID-19.^12^ The treatment with tocilizumab, a recombinant monoclonal antibody that binds specifically to IL-6 receptors, has been proposed as a promising strategy to control the virus-induced cytokine storm.^1-4^ However, literature concerning tocilizumab's ability to offset the cytokine storm and reduce the risk of in-hospital mortality in COVID-19 patients is severely lacking and remains to be validated in clinical trials.

Here we described a case series of three COVID-19 patients, who had received infusion/s of tocilizumab.

## CASE REPORTS

### CASE 1

**Figure 1:**
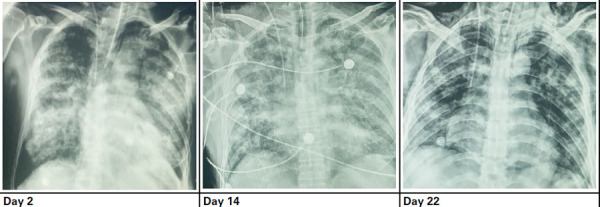
Chest radiograph images during the patient's hospitalisation.

A 76-year-old male, initially diagnosed with COVID-19 on 6 August 2021, was later brought to the COVID ward of on 8 August 2021 with chief complaints of fever, cough, and anosmia for 7 days. The patient had no history of hypertension, diabetes, or any other underlying diseases. He had taken both (first and second) doses of the Covishield vaccine. The patient was admitted to the high dependency unit (HDU) of NAPFH on 8 August 2021. On day 1, the patient was conscious with a blood pressure of 120/70 mmHg, pulse rate of 72 beats per minute, respiration rate of 22 breaths per minute, an axillary temperature of 36.2°C, and oxygen saturation (SpO_2_) level of 93%. Chest radiograph showed white-out bilateral lungs (Figure 1).

Routine laboratory tests showed elevated levels of C-reactive protein (CRP) (28 mg/l), glucose (324 mg/dl), urea (60 mg/dl), ALT (76 lU/l) and AST (88 lU/l). The patient was treated with dexamethasone 6 mg intravenous (IV) and low molecular weight heparin (LMWH) 60 U subcutaneous (s/c) every 12 hours for 3 days, remdesivir 200 mg (IV) on the first day, and 100 mg (IV) every 24 hours for 4 days, azithromycin 500 mg tablet every 24 hours for 5 days, meropenem 1 gm (IV) every 8 hours for 5 days, pantoprazole 40 mg (IV) every 24 hours for 3 days, levocetirizine 5 mg tablet every 12 hours for 3 days and other supportive medications, including tablets of zinc, vitamin C, paracetamol, and vitamin D capsules.

On day 11, the patient had higher blood pressure (160/80 mmHg) and respiration rate (28 breaths per minute), and lowered oxygen saturation level (90%). The patient was transferred to the Intensive Care Unit (ICU) and got additional therapy such as high flow nasal cannula oxygenation [Fraction of inspired O_2_ (FiO2): 100%], bilevel positive airway pressure (BiPAP), levofloxacin 750 mg every 24 hours for 5 days, unfractionated infusion of heparin for 1 day and dexamethasone 24 mg every 24 hours for 10 days. On day 12, laboratory examination revealed elevated levels of CRP (22.0 mg/l), procalcitonin (PCT) (0.1 ng/ml), IL-6 (340 pg/ml), white blood cells (WBC) (19,000 /μl), neutrophils (90%), platelets (328,000 /μl), D-dimer (3.4 mg/l) and lowered levels of albumin (2.8 g/dl) and alkaline phosphatase (ALP) (104 IU/l). The previously elevated serum biomarkers, such as glucose, urea, ALT, and AST remained elevated. Further, the levels of CRP (49 to >200 mg/l), PCT (0.2-25 ng/ml), D-dimer (0.7-4.1 mg/l), WBC (16,000-4,200 /μl) and platelets (238,000-110,000 /μl) substantially varied from day 13 to day 23. The patient had positive culture results for urine *(Candida albicans)* and endotracheal aspirate *(Klebsiella oxytoca).*

On day 23, the patient was treated with a single infusion of tocilizumab 400 mg. Even after the administration of tocilizumab (24-48 hours), the levels of IL-6 (1,800 pg/ml), CRP (>200 mg/l), PCT (3.1-1.6 ng/ml) and D-dimer (2.9-1.5 mg/l) did not lowered. Till day 27, the patient clinical and laboratory markers did not improve and the patient finally died because of refractory hypoxemia with septic shock.

### CASE 2

**Figure 2:**
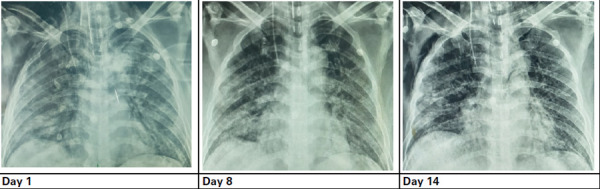
Chest radiograph images during the patient's hospitalisation.

A 31-year-old male, initially diagnosed with COVID-19 on 6 June 2021, was later brought to the COVID ward of NAPFH on 11 June, 2021 with chief complaints of fever, cough, hemoptysis, and dyspnea for a week. On the day of admission (11 June 2021), the patient had a lower level of SpO_2_ (65%), and hence, treatment was started with similar treatment protocols as patient 1 upon admission. On day 1, the patient was conscious with a blood pressure of 120/80 mmHg, pulse rate of 86 beats per minute, respiration rate of 24 breaths per minute, an axillary temperature of 36.6°C, and oxygen saturation (SpO_2_) level of 90%. The chest radiograph showed pneumonitis in the bilateral middle and lower zones (Figure 2).

Routine laboratory tests showed elevated levels of CRP (21.8 mg/l), WBC (11,300 /μl), neutrophils (85%), platelets (228,000 /μl), AST (59 IU/L), and lower level of ALP (60 IU/L). Additionally, the patient was treated with tablets of linezolid 600 mg and voriconazole 200 mg every 12 hours for 5 days. On day 2, the patient had a higher respiratory rate (28 breaths per minute) and severe COVID-19 pneumonia with acute respiratory distress syndrome (ARDS). Hence, the patient was admitted to the ICU, under mechanical ventilation support. The laboratory examination revealed elevated levels of CRP (65 mg/l), PCT (20.1 ng/ml), IL-6 (53 pg/ml), D-dimer (2.20 mg/l), prothrombin time (PT) (16 sec) and lowered levels of albumin (2 g/dl), ALP (58 lU/l) and hemoglobin (11.0 gm/dl). Further, the levels of WBC (11,400 /μl), neutrophil (88%), and platelet (189,000 /μl) remained elevated on day 3.

On day 4, the patient was treated with a single infusion of tocilizumab 400 mg. Despite the administration of tocilizumab, the levels of IL-6 (160 pg/ml), CRP (25 mg/l), ferritin (150 ng/ml), D-dimer (4.5 mg/l), WBC (14,800), and neutrophils (91%) did not varied significantly. The microbiological analysis revealed lower respiratory tract infection with *K. pneumonia,* indicating the occurrence of secondary bacterial infection (SBI) (≥48 hours of hospital admission). The condition of the patient did not improve, and hence the patient was subsequently treated with a second infusion of tocilizumab 400 mg on day 6. Initially, the therapy lowered the levels of CRP (32 mg/l), PCT (0.1 ng/ml), and platelets (165,000 /μl). However, the levels of CRP (18-55 mg/l), WBC (21,600-25,300 /μl), neutrophils (91-93%), D-dimer (5-7.2 mg/l), glucose (176-261 mg/dl), urea (81-136 mg/dl) and creatinine (1.6-2.4 mg/dl) kept on increasing from day 7 to 9. Ultimately, the patient developed severe sepsis with multiple organ dysfunction syndromes leading to his death.

### CASE 3

**Figure 3:**
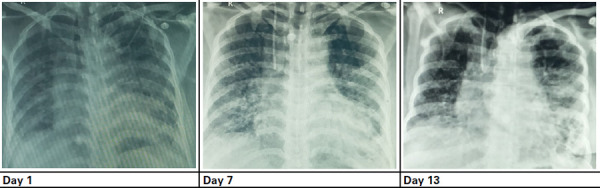
Chest radiograph images during the patient’s hospitalisation.

A 57-year-old male, initially diagnosed with COVID-19 on 7 May 2021, was later brought to the COVID ward of NAPFH on 25 May 2021 with chief complaints of cough and dyspnea for 21 days. The patient had a history of hypertension and was under medication. Upon hospitalization and admission to HDU, the patient was treated with similar treatment protocols as Patient 1. On day 1, the patient was conscious with a blood pressure of 110/70 mmHg, pulse rate of 82 beats per minute, respiration rate of 20 breaths per minute, an axillary temperature of 36.1°C, and oxygen saturation (SpO_2_) of 91%. Chest radiograph showed pneumonitis in the right middle and lower zone and left upper, middle, and lower zone (Figure 3).

Routine laboratory tests showed elevated levels of CRP (88 mg/l), WBC (17000 /μl), ALT (60 IU/1), and AST (63 lU/l). Additionally, the patient was treated with meropenem 1 gm (IV) every 8 hours for 5 days, budesonide 200 pg (puffing) every 12 hours for 3 days, and doxofylline 400 mg 12 hours for 3 days.

On day 7, the oxygen saturation level lowered (87%), and progressive dyspnea was observed. The patient was transferred to the ICU and got additional therapy, such as high-flow nasal cannula oxygenation (FiO2: 100%) and BiPAP intubation. On day 8, laboratory examination revealed elevated levels of WBC (18,200/μl), platelets (276,000 /μl), IL-6 (850 pg/mL), and lowered levels of albumin (3.0 g/dl) and total protein (5.6 lU/l). The previously elevated blood biomarkers, such as CRP (17 mg/l), neutrophils (90%), glucose (258 mg/dl), urea, ALT (83 lU/l), and AST (51 lU/l) remained elevated. On the same day, the patient was treated with a single infusion of tocilizumab 400 mg. After 24-48 hours, the patients' condition did not improve and the levels of IL-6 (820 pg/ml), CRP (32 mg/l), PCT (0.2 ng/ml), WBC (21,500 /μl), platelets (320,000 /μl), PT (15 sec), glucose (167 mg/dl), D-dimer (7.6 mg/l), urea (61 mg/dl), ALT (81 IU/L) and AST (79 IU/L) remained elevated. The patient had a positive culture result for urine *(C. albicans).* Hence, on day 10, the patient was subsequently treated with a second infusion of tocilizumab 400 mg. However, the levels of IL-6 (1,050 pg/ml), PCT (2.5 ng/mL), WBC (17,200 /μl), platelets (255,000/μl), D-dimer (5.8 mg/l), urea (170 mg/dl), ALT (86 IU/l) and AST (47 IU/L) did not subsided. Further, the levels of CRP (<5-116.0 mg/l), PCT (0.7-2.4 ng/ml), IL-6 (3,347 pg/ml), D-dimer (0.9-4.7 mg/l), prothrombin time (15 sec), WBC (8,000-14,000 /μl), platelets (200,000-80,000/μl), neutrophils (90-85%), urea (106171 mg/dl), ALT (31-54 IU/L), and ALP (56-69 IU/l) varied substantially from day 13 to day 18. The patient had positive culture results for sputum and endotracheal aspirate, by *Acinetobacter baumannii-calcoaceticus* complex and *Klebsiella pneumoniae*, respectively. On day 18, the patient's condition did not improve and the patient finally died because of type II respiratory failure with septicemia.

## DISCUSSION

Severe COVID-19 illness, which often requires admission of a patient to an ICU, is chiefly attributed to immune dysregulation.^2^ Activation of aberrant T cells and monocytes, which produce massive numbers of inflammatory cytokines, appears to be a significant factor for such dysregulation leading to cytokine storm.^2,3^ Multiple reports have identified a significant increase in the level of inflammatory markers, including IL-6, in patients with severe SARS-CoV-2 infection.^3,5,6^ Recently, the use of the IL-6 inhibitor antibody, tocilizumab, which can specifically bind to the soluble IL-6 receptor and inhibit signal transduction,^7^ has been utilized in the treatment of cytokine storm associated with COVID-19.^3^ It has been hypothesized that the decrease in endogenous levels of IL-6 using tocilizumab could lead to a reduction in cytokine and acute phase reactants producers and subsequently improves the clinical situation of the patient.^8^

The clinical response observed in the COVID-19 patients [mean age (±standard deviation): 54.7 years (±22.6); male: 100%] in this study following treatment with tocilizumab (400 mg/20ml) is in contrast with the previously published report.^3^ The indications for therapy in this study were decreased SpO_2_ (< 90%) level (100%), increased respiration rate (>16 breathes per minute) (100%), or both decreased SpO_2_ and increased respiration rate (100%), as per the guidelines of National Institute of Health.^9^ The patient's condition (100%) did not improve clinically after 48 hours of tocilizumab administration and had abnormal clinical, radiological, and laboratory markers indicating clinical deterioration. The IL-6 levels (n=2) remained significantly elevated (>1,000 pg/ml) even after tocilizumab administration, which could be due to delayed immune clearance and accumulation of IL-6 in the blood. Moreover, there was an increase in levels of CRP (>55 mg/l), PCT (>0.1 ng/mL), D-dimer (>0.9 mg/l), WBC (>20,000/μl), urea (>80 mg/dl), and a decrease in levels of platelets, albumin, and SpO_2_, following 72 hours of tocilizumab administration. Finally, all patients died; either because of septic shock or multiple organ dysfunction syndromes, and no benefit in mortality was seen. Though the patients were in a critical stage due to COVID-19, when interviewed with the patients or their relatives, they showed satisfaction with the overall treatment protocols of the treating clinicians. All patient recipient of tocilizumab infusion/s gave their approval for the treatment with tocilizumab, but with obvious concern for the high cost associated with the treatment with monoclonal antibody.

In contrast to this study, in an open-label trial in the United Kingdom that included 4,116 patients with suspected or confirmed COVID-19, hypoxemia (oxygen saturation <92% on room air or oxygen supplementation of any kind), and CRP level ≥75 mg/l, adding one or two doses of weight-based tocilizumab to usual care reduced the 28-day mortality rate compared to usual care alone (31% versus 35%, relative risk 0-85; 95% CI).^10^ Similarly, a retrospective analysis from two centres in China revealed that the use of 1-2 doses of tocilizumab in 21 adult patients made the patients afebrile with 70% decreased oxygen requirements and a higher hospital discharge rate.^11^ A retrospective analysis of 30 French patients with severe COVID-19 treated with tocilizumab after at least 5 prior days of illness requiring at least 6 l/min of oxygen therapy showed that treatment with tocilizumab 8 mg/kg for 1-2 doses could decrease ICU admission (p= 0.001) and mechanical ventilation (p= 0.025).^12^

Currently, there is no proven therapy to treat critically ill patients with COVID-19 other than supportive measures.^2^ The therapy with the IL-6 blockade in COVID-19 patients casts many questions regarding the optimal dose and timing of therapy.^2^ While a study has shown that early administration of tocilizumab before ICU admission could improve outcomes in COVID-19 patients,^13^ others have discussed a single infusion therapy as insufficient therapy.^2^ This case series documents that the treatment of COVID-19 patients with delayed administration of tocilizumab may not be effective in reducing the risk of in-hospital mortality, but may decline the elevated levels of inflammatory markers associated with COVID-19 cytokine storm.

Additionally, the adverse events upon tocilizumab administration, such as a transient decrease in leukocytes and an increased risk of bacterial infection in COVID-19 patients' remains a key concern.^7^

This case series had several limitations; having a small sample size and short-term follow-up. All patients received concomitant therapies, which may have changed the disease trajectory. Tocilizumab's therapy in a median of eight days from ICU admission in our study could have been too late to mitigate the lung damage and improve mortality. All observations were hypothesis-generating and cannot be considered causal.
